# Four-year safety and effectiveness data from patients with multiple sclerosis treated with fingolimod: The Spanish GILENYA registry

**DOI:** 10.1371/journal.pone.0258437

**Published:** 2021-10-13

**Authors:** J. E. Meca-Lallana, C. Oreja-Guevara, D. Muñoz, J. Olascoaga, A. Pato, L. Ramió-Torrentà, V. Meca-Lallana, M. A. Hernández, M. E. Marzo, J. C. Álvarez- Cermeño, A. Rodríguez-Antigüedad, X. Montalbán, O. Fernández, on behalf of the Spanish GILENYA Registry Investigators

**Affiliations:** 1 Neurology Department, Hospital Clínico Universitario Virgen de la Arrixaca, Murcia, Spain; 2 Neurology Department, Hospital Clínico San Carlos, Madrid, Spain; 3 Neurology Department, Hospital Xeral de Vigo, Vigo, Spain; 4 Neurology Department, Hospital Universitario Donostia, San Sebastián, Spain; 5 Neurology Department, Hospital Povisa, Vigo, Spain; 6 Neurology Department, Hospital Universitari de Girona Dr. Josep Trueta, IDIBGI; Medical Sciences Department, University of Girona, Girona, Spain; 7 Neurology Department, Hospital Universitario de La Princesa, Madrid, Spain; 8 Neurology Department, Hospital Universitario Nuestra Señora de Candelaria, Santa Cruz de Tenerife, Spain; 9 Hospital San Pedro, Logroño, Spain; 10 Neurology Department, IRYCIS, Hospital Universitario Ramón y Cajal, Madrid, Spain; 11 Neurology Department, Hospital Universitario Cruces, Barakaldo, Spain; 12 Neurology Department, Hospital Universitari Vall d’Hebron, Barcelona, Spain; 13 Department of Pharmacology, Faculty of Medicine, Universidad de Málaga; Instituto de Investigación Biomédica de Málaga (IBIMA), Málaga, Spain; University of Ioannina School of Medicine, GREECE

## Abstract

**Objective:**

To describe the profile of patients with multiple sclerosis (MS) treated with fingolimod in Spain and to assess the effectiveness and safety of fingolimod after 4 years of inclusion in the Spanish Gilenya Registry.

**Methods:**

An observational, retrospective/prospective, multicenter case registry, including all patients with relapsing-remitting MS (RRMS) starting treatment with fingolimod in 43 centers in Spain. Analyses were performed in the overall population and in subgroups according to prior disease-modifying therapy (DMT): glatiramer acetate/interferon beta-1 (BRACE), natalizumab, other treatment, or naïve.

**Results:**

Six hundred and sixty-six evaluable patients were included (91.1% previously treated with at least one DMT). The mean annualized relapse rate (ARR) prior to fingolimod was 1.12, and the mean EDSS at fingolimod initiation was 3.03. Fingolimod reduced the ARR by 71.4%, 75%, 75.5%, and 80.3%, after 1, 2, 3 and 4 years, respectively (p<0.001). This significant reduction in the ARR continued to be observed in all subgroups. After 4 years, the EDSS showed a minimal deterioration, with the EDSS scores from year 1 to year 4 remaining mostly stable. The percentage of patients without T1 Gd+ lesions progressively increased from 45.6% during the year prior to fingolimod initiation to 88.2% at year 4. The proportion of patients free from new/enlarged T2 lesions after 4 years of fingolimod treatment was 80.3%. This trend in both radiological measures was also observed in the subgroups. Adverse events (AEs) were experienced by up to 41.6% of patients (most commonly: lymphopenia [12.5%] and urinary tract infection [3.7%]). Most AEs were mild in severity, 3.6% of patients had serious AEs.

**Conclusions:**

The patient profile was similar to other observational studies. The results obtained from the long-term use of fingolimod showed that it was effective, regardless of prior DMT, and it had adequate safety results, with a positive benefit-risk balance.

## Introduction

Multiple sclerosis (MS) is an inflammatory demyelinating disease of the central nervous system (CNS) characterized by a wide range of symptoms [1]. MS is one of the world’s most common neurologic disorders, and its prevalence is increasing, with worldwide estimations rising from 2.3 million people in 2013 [2] to 2.8 million people in 2020 [3]. The number of available disease-modifying treatments (DMT) has greatly expanded in recent years, offering new challenges and opportunities to individualize treatment [4]. Fingolimod was the first oral DMT for MS approved by the European Medicines Agency (EMA) in 2011. It is a sphingosine 1-phosphate receptor modulator involved in immune cell trafficking that impedes lymphocyte from exiting lymph nodes into the circulation, preventing CNS inflammation [5]. Fingolimod is administered in a once daily dose and is indicated for patients with highly active relapsing-remitting multiple sclerosis (RRMS previously treated with at least one other DMT or with rapidly evolving severe disease [6].

The safety and efficacy of fingolimod were demonstrated in three double-blind, randomized phase 3 clinical trials: FREEDOMS I [7], FREEDOMS II [8] and TRANSFORMS [9]. The first two trials compared the safety and efficacy of fingolimod (0.5 mg and 1.25 mg) with placebo for 24 months, and the latter conducted these comparisons with intramuscular interferon beta-1a for 12 months. Fingolimod reduced the annualized relapse rate (ARR) and MRI outcomes (number of new/enlarged lesions on T2-weighted images, gadolinium-enhancing [Gd+] lesions, and brain-volume loss) [7,8], and the risk of disability progression [9]. Key eligibility criteria for patients in these trials were one or more documented relapses in the previous year or two or more in the previous two years, among others [7–9]. The characteristics of patients included in these pivotal clinical trials may differ from the eligible population in the clinical practice setting. In fact, a recent study has shown that 83% of patients receiving a DMT in routine clinical practice did not match selection criteria of the respective phase 3 clinical trials [10]. Thus, it is necessary to assess the safety and effectiveness of fingolimod outside the clinical trial setting in a more heterogenous patient profile.

While several real-word studies have provided clinical experience with fingolimod in daily practice [11–20], these studies were conducted in small populations [12,14,15,17–20] had a retrospective design [12–14,16,20], a limited follow-up period [17,18], or did not analyze the data considering patient subgroups treated with different prior DMT and treatment-naïve patients [11,12,14–18,20]. Moreover, none of these studies were patient registry-based. The important role of registry-based studies in the generation of post-authorization safety and effectiveness evidence in a broader clinical context and a more heterogenous patient population than randomised controlled trials has been emphasized by the EMA [21].

The Spanish Gilenya registry was designed to assess patients characteristics and long-term safety and effectiveness of fingolimod in patients treated in Spanish clinical practice [22]. Preliminary analysis for patients without prior DMT for MS from this registry has been published, and supported the use of fingolimod in these patients [23]. However, long-term data for all the patients included in the registry remained unexplored. Here, we present the profile of patients treated with fingolimod over 4 years with and without prior DMT that were included in the Spanish Gilenya registry. and the results for safety and effectiveness of fingolimod over this time period.

## Materials and methods

### Ethics statement

The study protocol was classified by the Spanish Agency for Medicines and Health Products and received a favorable ethical opinion from the Ethics Committee at the Hospital Universitario La Paz (Madrid).

The study was conducted following a priori defined methods and in accordance with the Declaration of Helsinki. The study is reported as per the Strengthening the Reporting of Observational Studies in Epidemiology (STROBE) guideline. Written informed consent was obtained from all participants before their inclusion in the study.

### Study design and setting

This was an observational, retrospective/prospective, multicenter case registry in RRMS patients starting treatment with fingolimod. Patients were recruited from Neurology departments and MS Units of 43 participating centers in Spain between May 2012 and April 2016. Fingolimod was dispensed according to standard clinical practice in Spain and to the European fingolimod summary of product characteristics (SmPC). All patients initiated fingolimod at the baseline visit. Follow-up visits occurred at month 6, year 1, year 2, year 3, and year 4, with a time window of ±90 days for each visit. Data were recorded in standardized electronic case report forms.

### Participants

Patients were included in the study if they: i) were 18 years or older; ii) had been diagnosed with RRMS; iii) were treated or planned to be treated with fingolimod; iv) attended a Neurology department or MS unit; v) gave informed consent. Patients who were receiving fingolimod as part of a clinical trial were excluded from the study.

### Outcome measures

Demographic (gender, age) and clinical characteristics (disease duration, anti-JC virus antibodies, Expanded Disability Status Scale [EDSS] score, prior MS treatments), anthropometric measurements, and smoking habit were collected. Effectiveness outcome measures were: disability outcomes using the EDSS scores after 6 months of fingolimod initiation and annually, proportion of patients free from relapses the year before fingolimod initiation and each subsequent year, radiological disease activity status (Gd+ T1 lesions and new/enlarged T2 lesions) the year before fingolimod initiation (±90 days) and each following year (±90 days), proportion of patients free from disease activity (clinical, radiological, and clinical and radiological activity) each year after fingolimod initiation, and proportion of patients who needed concomitant treatment after fingolimod initiation. Patients free from clinical activity were defined as those without relapses and with improvements or stability in the EDSS score. Patients free from radiological activity were defined as those without T1 Gd+ lesions and new/enlarged T2 lesions. Patients free from clinical and radiological activity were defined as those without clinical activity and radiological activity considering the definition above. Safety was assessed by the proportion of patients experiencing adverse events (AEs) and discontinuation of fingolimod treatment due to an AE.

### Statistical analysis

All patients meeting selection criteria with minimum demographic data (gender or age) and information on the date of fingolimod treatment initiation or under follow-up after receiving the first dose were included in the analyses. Safety analyses were conducted in all patients meeting selection criteria with any follow-up data.

Baseline characteristics and effectiveness analyses were performed for the overall study population and stratified into four subgroups based on the use of DMTs prior to fingolimod initiation: those who had not received any DMT (naïve), those who received natalizumab (prior-NTZ), those who received BRACE therapies (Betaseron [interferon beta-1b sc], Rebif [interferon beta-1a sc], Avonex [interferon beta-1a im], Copaxone [glatiramer acetate], and Extavia [interferon beta-1b sc]) (prior-BRACE), and those who received other DMTs (prior-Other). Safety analyses were performed for the overall study population.

Data for categorical variables are presented as the number and proportion of cases in each category. For continuous variables, data are summarized using means, standard deviations (SD), medians, and interquartile ranges (IQR). Quantitative variables were compared using the nonparametric Kruskal-Wallis test. Qualitative variables were compared using Fisher’s exact test. All statistical tests were two-sided at a significance level of 0.05. No imputations for missing data were made. The statistical analysis was performed using SAS version 9.4 software.

## Results

A total of 702 patients consented to participate in the study, of which 697 patients were included in the safety analysis and 666 had the minimum information to be included in the baseline characteristics and effectiveness analysis.

### Baseline characteristics

Table 1 shows the demographic and clinical characteristics at baseline of the overall MS population and of each subgroup according to the prior DMT received. Briefly, women were predominant in the overall patient population (71.1%), with a mean (SD) age of 39.1 (8.8) years. The mean (SD) time since diagnosis was 9.0 (6.1) years. The mean (SD) fingolimod treatment duration was 38.3 (16.7) months. Prior to fingolimod initiation, 91.1% (n = 612) of patients were previously treated with at least one DMT, the most common DMT being natalizumab (33.5%), followed by glatiramer acetate (23.4%) and interferon beta-1a (17.0%). There were significant differences between prior DMT subgroups in terms of age, time since first MS symptoms and time since diagnosis: naïve patients were younger (p = 0.026), had shorter MS symptoms (p<0.001) and shorter disease duration (p<0.001). Differences in the mean (SD) EDSS score at baseline were also observed between subgroups (2.5 [1.7] naive; 2.8 [1.6] prior-BRACE; 3.5 [1.8] prior-NTZ; 3.9 [1.9] prior-Other; p<0.001). The rest of the demographic and clinical characteristics at baseline were similar across the subgroups.

**Table 1:** Demographic and clinical characteristics at baseline of the overall population and prior treatment subgroups.

Characteristics	Overall population (n = 666)	Naïve (n = 54)	Prior-BRACE (n = 376)	Prior-NTZ (n = 205)	Prior-Other (n = 31)	P value
Age (years), mean ± SD	39.1±8.8	36.2±8.4	39.1±8.7	39.2±8.7	42.2±9.5	0.026
Female, n (%)	472 (71.1)	38 (70.4)	265 (70.5)	146 (71.9)	23 (74.2)	0.974
BMI (kg/m^2^), mean ± SD	24.0±4.1	24.5±6.0	24.3±4.0	23.6±4	22.3±3.6	0.084
Smokers, n (%)	154 (27.7)	12 (30.2)	91 (28.4)	43 (25.8)	7 (25.9)	0.882
Time since first MS symptoms (years), mean ± SD	11.2±7.0	4.9±5.8	10.8±6.4	12.9±6.8	14.5±9.0	<0.001
Time since diagnosis (years), mean ± SD	9.0±6.1	2.8±5.5	8.8±5.9	10.6±5.5	11.5±7.1	<0.001
EDSS at fingolimod initiation						
Mean ± SD (n)	3.03±1.73 (n = 556)	2.49±1.72 (42)	2.80±1.63 (n = 315)	3.45±1.77 (n = 174)	3.94±1.92 (n = 25)	<0.001
Median (IQR)	3.0 (2.0)	2.5 (2.0)	2.5 (2.5)	3.5 (2.5)	4.0 (3.0)	
Anti-JC Virus antibodies presence, n (%)	382 (86.0)	29 (93.6)	170 (82.1)	171 (89.5)	12 (80.0)	0.076

*Abbreviatures*: BMI, Body Mass Index; BRACE: Betaseron (interferon beta-1b sc), Rebif (interferon beta-1a sc), Avonex (interferon beta-1a im), Copaxone (Glatiramer acetate), and Extavia (interferon beta-1b sc); EDSS, Expanded Disability Status Scale; IQR, interquartile range; NTZ, natalizumab; SD, standard deviation.

### Effectiveness

#### Relapses

**Fig 1:**
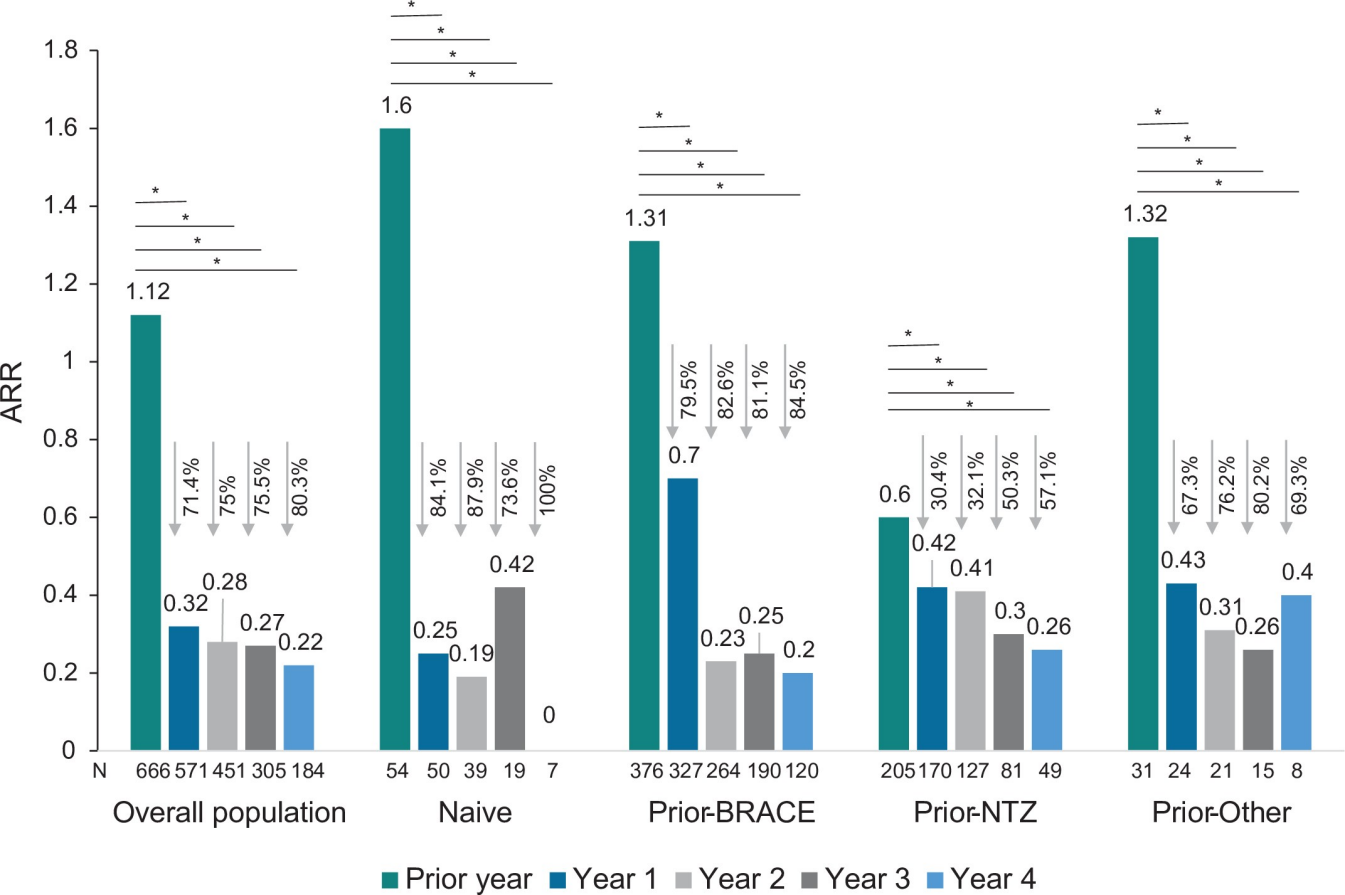
ARR in the overall population and prior treatment subgroups. *Abbreviatures*: BRACE: Betaseron (interferon beta-1b sc), Rebif (interferon beta-1a sc), Avonex (interferon beta-1a im), Copaxone (Glatiramer acetate), and Extavia (interferon beta-1b sc); EDSS, Expanded Disability Status Scale; IQR, interquartile range; N: Number of patients with available data for relapses; NTZ, natalizumab.

The ARR in the overall population decreased significantly from 1.12 the year prior to fingolimod initiation to 0.32 at year 1, 0.28 at year 2, 0.27 at year 3 and 0.22 at year 4, representing a reduction of 71.4%, 75%, 75.5%, and 80.3%, respectively (p<0.001). The decreases in the ARR were also significant in all subgroups of patients at every follow-up (see Fig 1), including in the prior-NTZ subgroup, which had a reduction of 57.1% at year 4 (0.26) compared to the year prior to fingolimod initiation (0.60) (p = 0.003).

The proportion of patients free from relapses after fingolimod initiation was 75.5% at year 1, 78.3% at year 2, 80.0% at year 3, and 84.2% at year 4, in the overall population. The proportion of patients free from relapses in the visit prior to fingolimod initiation was significantly different among subgroups (p<0.001, with a higher proportion of patients free from relapses in the prior-NTZ subgroup (65.9%) than in the other subgroups (16.7% naïve; 15.7% prior-BRACE; 17.1% prior-Other). No significant differences in the proportion of patients free from relapses between subgroups were found either at year 1 (p = 0.355), year 2 (p = 0.066), year 3 (p = 0.079), or year 4 (p = 0.277).

#### Disability

**Fig 2:**
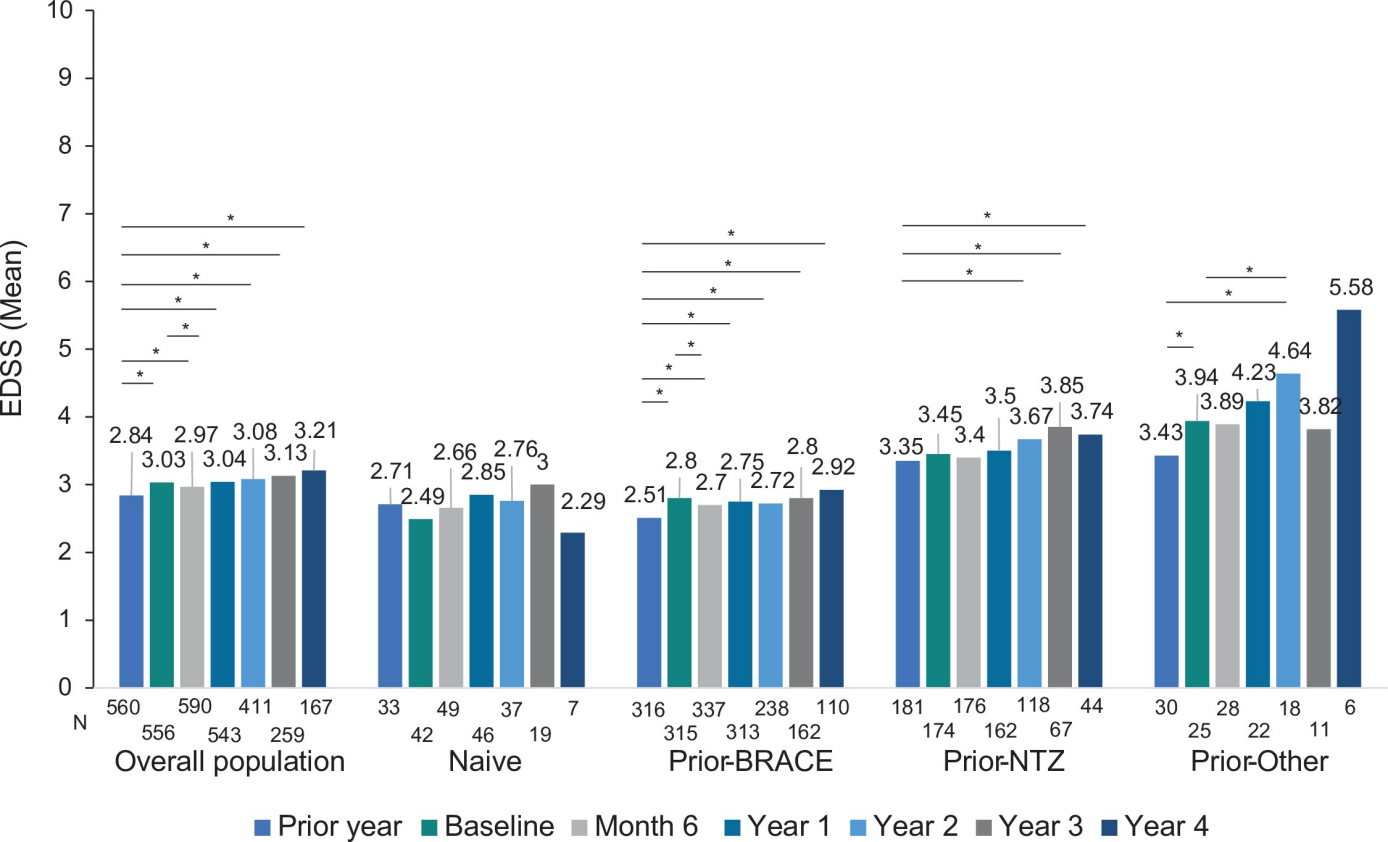
Expanded Disability Status Scale evolution in the overall population and prior treatment subgroups. *Abbreviatures*: BRACE: Betaseron (interferon beta-1b sc), Rebif (interferon beta-1a sc), Avonex (interferon beta-1a im), Copaxone (Glatiramer acetate), and Extavia (interferon beta-1b sc); EDSS, Expanded Disability Status Scale; IQR, interquartile range; N: Number of patients with available data for EDSS at that visit; NTZ, natalizumab. *Significant (p<0.05) paired comparation EDSS at month 6, year 1, year 2, year 3, and year 4 vs year prior to fingolimod and vs baseline are shown. Comparisons have been conducted considering the patients with EDSS data at both visits. Not significant comparisons are not marked.

The mean (SD) EDSS score of the overall population at the visit the year prior to fingolimod initiation was 2.84 (1.79), which was lower than the EDSS score at the time of fingolimod initiation (baseline) (3.03 [1.73]; p<0.001). Lower EDSS scores the year prior to fingolimod initiation than at baseline were also observed in prior-BRACE (2.51 [1.69] vs 2.80 [1.63]; p<0.001) and other-DMT (3.43 [2.01] vs 3.94 [1.92]; p = 0.003) subgroups, but not in naïve (2.71 [1.85] vs 2.49 [1.72]; p = 0.714) and prior-NTZ (3.35 [1.77] vs 3.45 [1.77]; p = 0.727) subgroups (see Fig 2). In the overall population, the mean EDSS (SD) score at each follow-up (2.97 [1.73] at month 6; 3.04 [1.91] year 1; 3.08 [2.01] year 2; 3.13 [2.01] year 3; and 3.21 [2.01] year 4) was higher than the EDSS score at the visit prior to fingolimod initiation. However, when the mean EDSS (SD) score at each follow-up was compared to baseline (3.03 [1.73]), a lower EDSS score was only observed at month 6 (2.97 [1.86]; p = 0.004), while EDSS scores from year 1 to year 4 remained stable. Lower EDSS scores compared to the visit the year prior to fingolimod initiation were also observed in the prior-BRACE subgroup at all visits (2.51 [1.69] in the year prior vs 2.70 [1.77] at month 6 [p = 0.005]; 2.75 [1.83] at year 1 [p = 0.001]; 2.72 [1.95] at year 2 [p<0.001]; 2.80 [2.04] at year 3 [p = 0.004]; and 2.92 [1.99], at year 4 [p = 0.051]) and in the prior-NTZ from year 2 to year 4 (3.35 [3.35] in the year prior vs 3.67 [1.92] at year 2 [p = 0.025]; 3.85 [1.81] at year 3 [0.019]; and 3.74 [1.89] at year 4 [p = 0.046]).

#### Radiological activity

**Fig 3:**
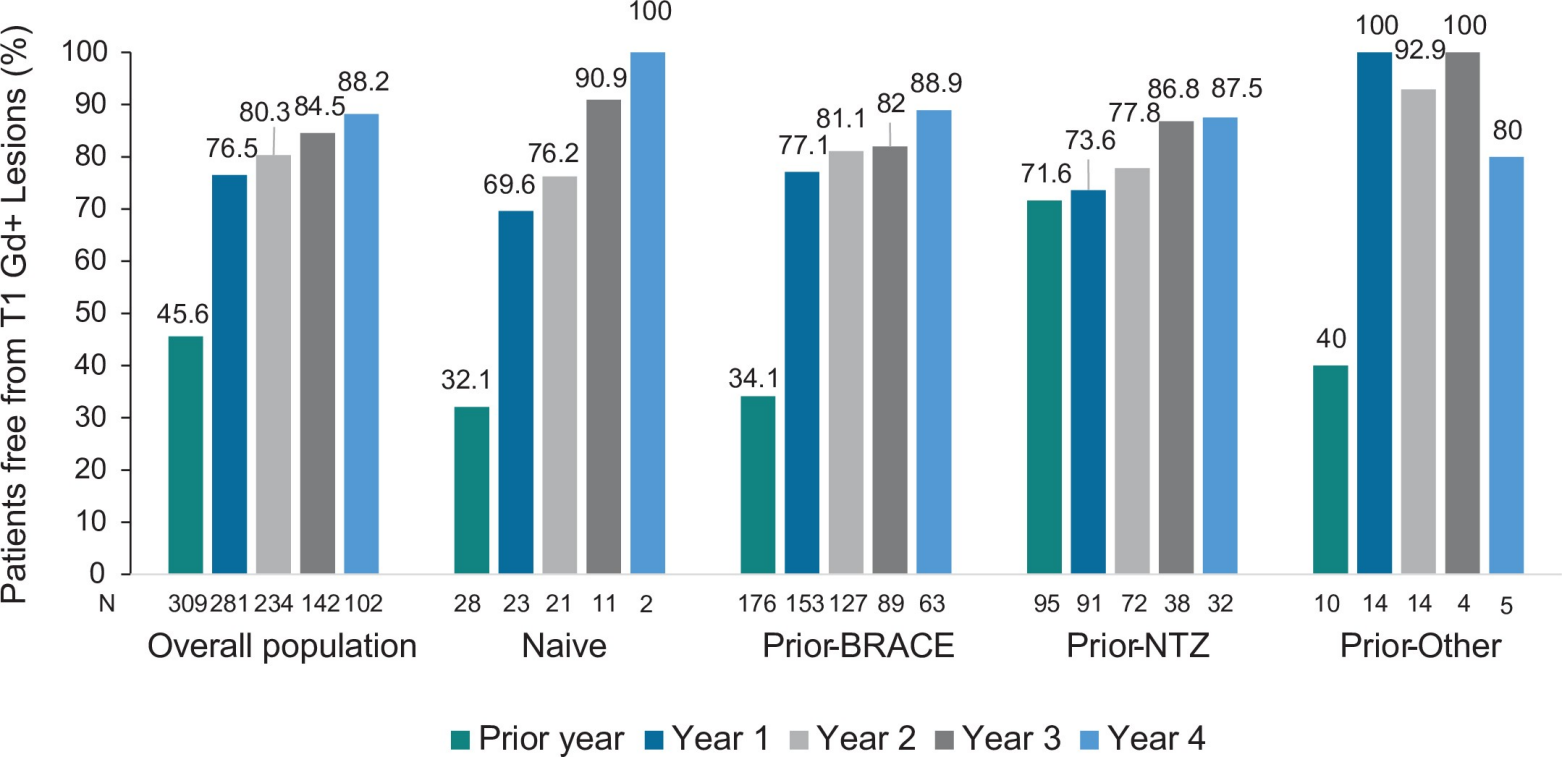
Patients free from T1 Gd+ Lesions in the overall population and prior treatment subgroups. *Abbreviatures*: BRACE: Betaseron (interferon beta-1b sc), Rebif (interferon beta-1a sc), Avonex (interferon beta-1a im), Copaxone (Glatiramer acetate), and Extavia (interferon beta-1b sc); EDSS, Expanded Disability Status Scale; IQR, interquartile range; N: Number of patients with available data for T1 Gd+ Lesions; NTZ, natalizumab.

The percentage of patients without T1 Gd+ lesions progressively increased from 45.6% during the year prior to fingolimod initiation to 88.2% at year 4 in the overall population. This increase was also observed in naïve, prior-BRACE, and prior-NTZ patients (see Fig 3). Despite the percentage of patients free from Gd+ lesions the year before fingolimod treatment being significantly higher in patients with prior-NTZ (71.6%) compared to naïve (32.1%), prior-BRACE (34.1%) or other-DMT patients (40%) (p<0.001), differences between subgroups disappeared after fingolimod treatment initiation (p = 0.095 at year 1; p = 0.589 at year 2; p = 0.848 at year 3; and p = 0.728 at year 4).

**Fig 4:**
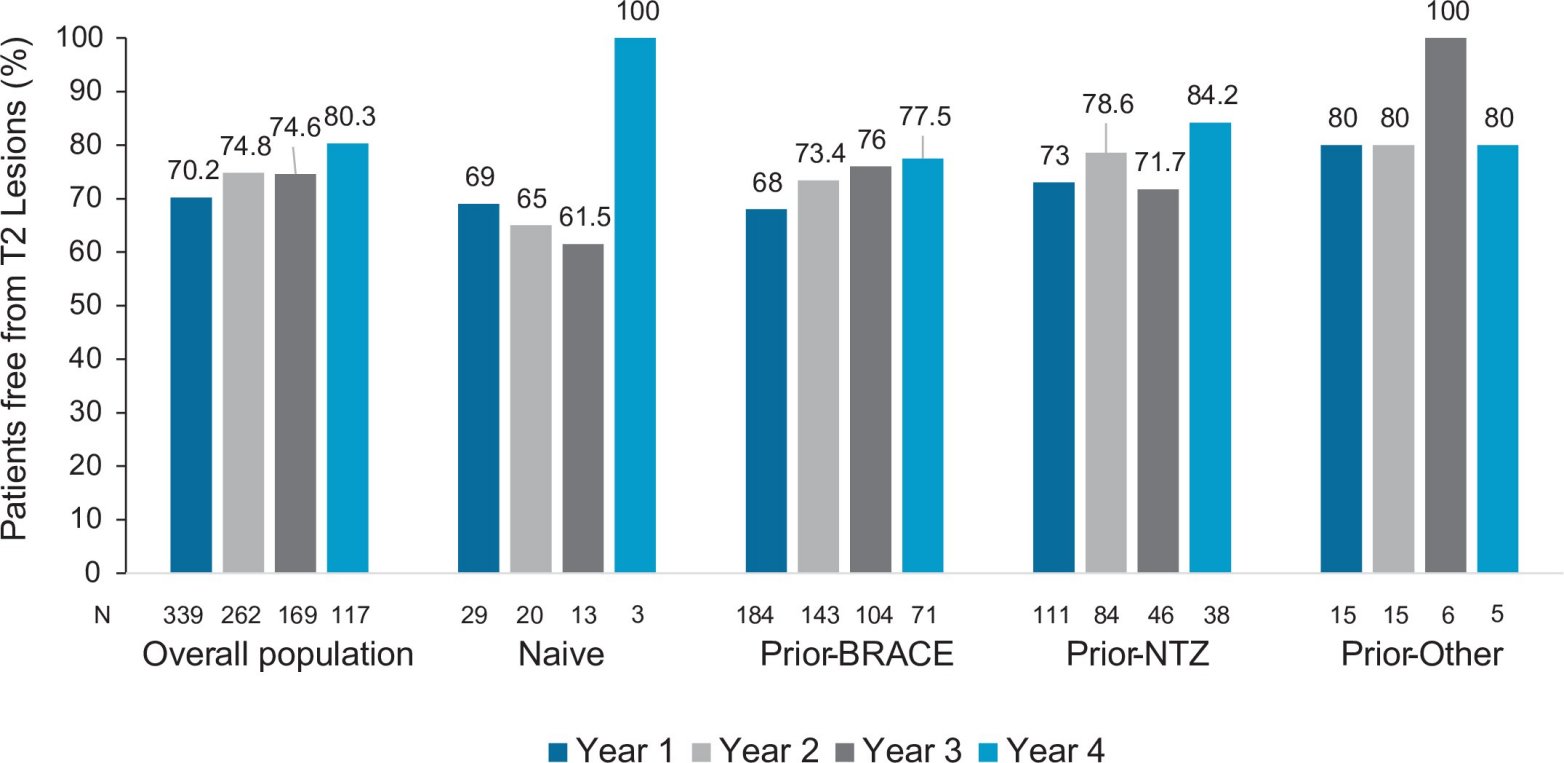
Patients free from new/enlarged T2 lesions in the overall population and prior treatment subgroups. *Abbreviatures*: BRACE: Betaseron (interferon beta-1b sc), Rebif (interferon beta-1a sc), Avonex (interferon beta-1a im), Copaxone (Glatiramer acetate), and Extavia (interferon beta-1b sc); EDSS, Expanded Disability Status Scale; IQR, interquartile range; N: Number of patients with available data for T2 Lesions; NTZ, natalizumab.

A total of 70.2% and 80.3% of patients were free from new/enlarged T2 lesions after 1 and 4 years of fingolimod treatment, respectively. The increasing percentage of patients free from new/enlarged T2 lesions from year 1 to year 4 was also observed in patients previously treated with BRACE (68% at year 1; 77.5% at year 4), NTZ (73% at year 1; 84.2% at year 4) or naïve patients (69% at year 1; 100% at year 4), although in the latter the number of patients at year 4 was only 3 (see Fig 4). No differences between subgroups were observed at any visit during year 4 of the follow-up.

#### Disease activity

**Fig 5:**
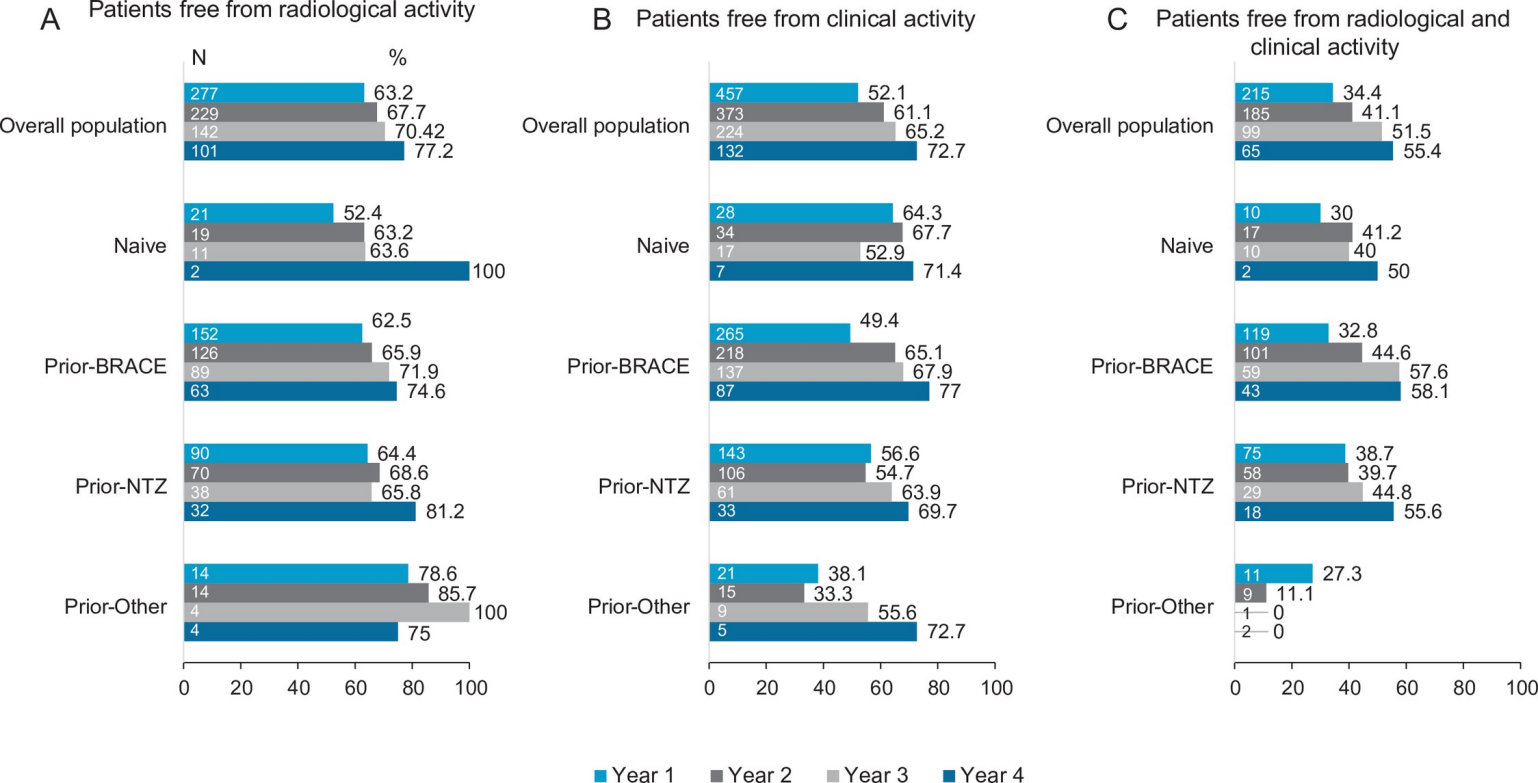
MS activity after 4 years vs prior year of fingolimod treatment in the overall population and prior treatment subgroups. *Abbreviatures*: BRACE: Betaseron (interferon beta-1b sc), Rebif (interferon beta-1a sc), Avonex (interferon beta-1a im), Copaxone (Glatiramer acetate), and Extavia (interferon beta-1b sc); EDSS, Expanded Disability Status Scale; IQR, interquartile range; N: Number of patients with available data for radiological (A), clinical (B), or radiological and clinical (C) activity; NTZ, natalizumab.

Fig 5 shows the percentage of patients free from clinical (section A), radiological (section B), and clinical and radiological (section C) activity for the overall population and for each subgroup. Considering the overall population, an increase in the percentage of patients at each visit without clinical activity (63.2% at year 1; 67.7% at year 2; 70.4% at year 3; and 77.2% at year 4), radiological activity (52.1% at year 1; 61.1% at year 2; 65.2% at year 3; and 72.7% at year 4) and clinical and radiological activity (34.4% at year 1; 41.1% at year 2; 51.5% at year 3; and 55.4% at year 4) was observed. No differences between subgroups were detected, except for the percentage of patients free from clinical activity at year 2 (67.7% naïve; 65.1% prior-BRACE; 54.7% prior-NTZ; and 33.3% prior-Other; p = 0.033).

#### Concomitant treatments

Since fingolimod treatment initiation, 46.1% of patients had concomitant medication for the nervous system. Patients also received concomitant medication for the cardiovascular system (18.2%), alimentary tract and metabolism (17.9%), genitourinary system and sex hormones (15.2%), and musculoskeletal system (15%), among others. Details on the percentage of patients with concomitant medication after fingolimod initiation in the overall population and by each subgroup are displayed on Table 2.

**Table 2:** Patients with concomitant medication after fingolimod initiation.

	Overall population (n = 666)	Naïve (n = 54)	Prior-BRACE (n = 376)	Prior-NTZ (n = 205)	Prior-Other (n = 31)
	**% (n)**				
Nervous system	46.1 (307)	44.4 (24)	44.4 (167)	49.8 (102)	45.2 (14)
Cardiovascular system	18.2 (121)	16.7 (9)	17 (64)	20 (41)	22.6 (7)
Alimentary tract and metabolism	17.9 (119)	20.4 (11)	16.8 (63)	18.1 (37)	25.8 (8)
Genitourinary system and sex hormones	15.2 (101)	11.1 (6)	12 (45)	20.5 (42)	25.8 (8)
Musculoskeletal system	15 (100)	5.6 (3)	12.2 (46)	22 (45)	19.4 (6)
Blood and blood-forming organs	7.2 (48)	5.6 (3)	5.1 (19)	11.2 (23)	9.7 (3)
Dermatological	6 (40)	7.4 (4)	3.5 (13)	10.7 (22)	3.2 (1)
Anti-infectives for systemic use	4.2 (28)	7.4 (4)	2.9 (11)	6.3 (13)	0 (0)

*Abbreviatures*: BRACE: Betaseron (interferon beta-1b sc), Rebif (interferon beta-1a sc), Avonex (interferon beta-1a im), Copaxone (Glatiramer acetate), and Extavia (interferon beta-1b sc); EDSS, Expanded Disability Status Scale; IQR, interquartile range; NTZ, natalizumab.

### Safety

The first fingolimod dose was monitored for a mean (SD) of 7.2±6.6 hours. During the 30 days after administration of the first dose, adverse reactions were reported by 7.5% of patients, the most common being grade 4 (<200 cells/μl) lymphopenia (3.0%), followed by bradycardia (1.2%).

During the study period, a total of 290 (41.6%) patients (out of the 697 patients in the safety sample) experienced at least one adverse event during fingolimod treatment. Most of the AEs were considered to be mild in severity, with 3.6% of patients experiencing serious AEs (SAEs). The most commonly reported AEs were grade 4 lymphopenia (12.5%) and urinary tract infection (3.7%). Table 3 summarizes AEs reported for ≥1% of the patients of the overall population.

**Table 3:** Adverse events for the overall population (reported in ≥1% of patients).

	Overall population (n = 697)	
	Number (%) of patients with AEs	Number of AEs
Blood and lymphatic system disorders	92 (13.2)	101
Lymphopenia (Grade 4)	87 (12.5)	96
Infections and infestations	86 (12.2)	120
Urinary tract infection	27 (3.7)	35
Candida infection	10 (1.3)	10
Upper respiratory tract infection	9 (1.3)	9
Nervous system disorders	51 (7.2)	65
Headache	19 (2.7)	20
Hepatobiliary disorders	25 (3.6)	26
Hypertransaminasemia	15 (2.1)	16
Skin and subcutaneous tissue disorders		21
Alopecia	21 (3.0)	7
	7 (1.0)	
General disorders and administration site conditions	18 (2.6)	19
Cardiac disorders	17 (2.4)	17
Bradycardia	9 (1.3)	9
Psychiatric disorders	17 (2.4)	18
Insomnia	7 (1.0)	7
Investigations	16 (2.3)	17
increased transaminases	13 (1.9)	14
Musculoskeletal and connective tissue disorders	15 (2.1)	20
Gastrointestinal disorders	14 (2.0)	15
Neoplasms benign, malignant and unspecified (incl. cysts and polyps)	10 (1.4)	10
Eye disorders	7 (1.0)	8

*Abbreviatures*: AEs, adverse events.

An adverse drug reaction occurred in 25.1% of patients between fingolimod initiation and the follow-up visit at year 4 (S1 Table). In 9 patients (1.3%), these adverse drug reactions were severe.

After the 4-year follow-up period, a total of 6.5% of patients temporarily interrupted treatment and 15.9% of patients discontinued (see S2 Table). The most frequently reported reasons for treatment interruption were clinical criteria (5.3%), followed by pregnancy or desire for pregnancy (1.6%), and persistent lymphopenia (0.5%). No deaths occurred during the study. S2 Table presents the proportion of patients who temporarily interrupted or discontinued treatment each year.

## Discussion

The Spanish Gilenya Registry provides information on the patient profile, effectiveness and safety of fingolimod in MS patients treated in routine care during a 4-year follow-up. Observational studies, using an electronic case registry design as in the present study, allow us to adequately reassess in a real-world MS population the benefit–risk profile of DMTs observed in randomized clinical trials. Our findings add to the growing body of evidence that the use of fingolimod in RRMS patients is associated with sustained effectiveness and a manageable safety profile [11,14,17,19,20,24,25]. The novelty and strength of the present study lies in the fact that, to our knowledge, it is one of the first studies reporting data from patients treated with fingolimod stratified according to prior treatment with a follow-up of 4 years. This is a longer follow-up than in previous prospective studies, including both observational studies [11,26,27] and clinical trials [7,9], or the observational period in retrospective studies which has usually been 1 or 2 years [14,24,25,28], with a maximum follow-up of 3 years [11].

### Patient profile

Compared to the FREEDOMS and TRANSFORMS clinical trials [7,9], patients in our study were older (39.1 years old vs 36.6 in FREEDOMS and 36.7 in TRANSFORMS), had higher EDSS scores (3.03 vs 2.3 in FREEDOMS and 2.24 in TRANSFORMS), and a higher proportion of patients had received at least on prior DMT (91.1% vs 42.6% in FREEDOMS and 55.2% in TRANSFORMS). However, when these baseline characteristics are compared with other real-world evidence (RWE) studies, the profile in terms of age, EDSS, or prior DMT use [11,14,24–27] is similar, although older patients and higher EDSS scores were also reported [28]. These differences in patient profile between the pivotal trials and RWE studies reinforce the importance of conducting studies in more realistic conditions to complement clinical trial data. In fact, a recent study has shown that only 27.7% of MS patients treated with fingolimod in routine clinical practice would have been eligible for the fingolimod phase III clinical trials [29].

### Effectiveness in the overall population and by prior treatment

Our findings confirmed fingolimod effectiveness in reducing relapse activity seen in pivotal trials [7,9]. The ARR after 1 year of treatment (0.32) was similar to those reported in other RWE studies (0.28 [24], 0.31 [26], 0.32 [27,28]) and between the range of other lower (0.23 [14]) and higher (0.42 [30]) ARR findings. Notably, the ARR continued to decrease with each year of fingolimod treatment. The ARR at year 3 (0.27) was almost identical to the ARR observed at month 36 in the PANGAEA study (0.265) [11]. The proportion of patients remaining free from relapses also progressively increased every year, achieving an 84.2% of patients free from relapses at year 4. This finding indicates that fingolimod effectiveness is sustained in the long-term. It also suggests that the benefits of fingolimod on relapse occurrence may increase with duration, although this hypothesis cannot be confirmed with the present data because the number of patients at year 4 had considerably decreased from the beginning of the study and the remaining patients may represent those in whom fingolimod was more effective, leading to a responder bias.

Importantly, the benefits on the ARR were present regardless of the prior treatment, including patients who had switched from natalizumab to fingolimod. The effectiveness of fingolimod in reducing relapses in this subpopulation has been heterogeneous. One retrospective study conducted in Spain showed that in patients previously treated with natalizumab, the ARR did not improve from the 24 months prior to fingolimod treatment (0.3) to the first 24 months of fingolimod (0.2) [25]. Another Spanish study, with a 2-year prospective follow-up, found that the ARR at year 1 (0.29) increased compared to baseline (0) with a subsequent decrease at year 2 (0.16) in patients who switched from natalizumab to fingolimod due to risk of developing progressive multifocal leukoencephalopathy [26]. Our results of a significant ARR reduction of 30.4% at year 1 in the prior natalizumab group are between the range reported by Galan Sánchez-Seco et al. (23.5%) [31] and Mazibrada et al. (41%) [28]. The ARR at year 1 (0.42) and year 2 (0.41) in our study was also similar to the ARR over 48 months of fingolimod treatment reported by Ziemssen et al. (which ranged between 0.45 and 0.54 depending on washout durations) [32].

The EDSS showed a minimal deterioration after 4 years. Our data is in line with the results from the PANGAEA study, where patients also had a mean baseline EDSS of 3.0 and remained stable over 4 years [30]. Despite EDSS scores remaining stable in patients previously treated with natalizumab and with other treatments, their scores were higher, probably due to a more aggressive course or advanced state of disease in these subpopulations than in naïve or prior-BRACE subgroups. Higher EDSS in patients previously treated with natalizumab have also been observed in other studies, both at baseline and during fingolimod treatment [14,24,26,31]. Fingolimod has been shown to be less effective when the EDSS score at baseline is above 3 [25,26]. Our data support the idea that fingolimod could be a more appropriate DMT in earlier forms of the disease when patients have less disability.

In terms of radiological activity, a high proportion of patients remained free from T1 Gd+ lesions and new/enlarged T2 lesions up to year 4. Compared with other Spanish RWE studies, our data showed that the percentage of patients free from T1 Gd+ lesions increased every year, while the opposite was observed by Izquierdo et al. [25]. The percentage of patients free from new/enlarged T2 lesions was comparable to that found by Barrero et al. [24] at year 1 and 2, but it was higher at year 3. It is worth mentioning that fingolimod benefits on radiological activity were observed regardless of the prior treatment. At year 1, the percentage of patients free from radiological activity (63.2% in the overall population, 65.9% in prior-BRACE, and 64.4% in prior-NTZ) was more homogeneous than those reported previously (72.3% in the overall population, 81.1% in prior-BRACE, and 68% in prior-NTZ) [14]. The fact that the percentage of prior-NTZ patients free from radiological activity at year 1 was similar to the prior-BRACE subgroup suggests an earlier benefit of fingolimod treatment on radiological activity in the former subgroup than previously shown [26].

### Safety profile

One of the advantages of registry-based studies is the assessment of AEs in a heterogeneous patient population, who have comorbidities, concomitant medications, and other idiosyncrasies. In our study, 41.6% of patients had at least one AE during fingolimod treatment. This number is lower than the percentage reported in the pivotal clinical trials (86% [9], 94% [26]), and lies between the 20%-72% range previously reported in the real-world setting ([11,12,14,24–26,33–35]). The incidence of SAEs was low (3.6%), consistent with available long-term safety data (3.9%) [11]. Among these AEs and SAEs, only 25.1% and 1.3%, respectively, were considered to be related to fingolimod. The nature of these adverse drug reactions was in line with the list included in the SmPC [36], although some differences were observed. Certain adverse drug reactions cited as very common in the SmPC (influenza, sinusitis, cough, diarrhea, and back pain) were not common or even present among our patients. The most common adverse drug reactions in the registry were lymphopenia and infections, which have been also commonly observed in other post-authorization studies [26,35]. Rare cases of progressive multifocal leukoencephalopathy [37] and cryptococcal infections [38] were not detected here.

Treatment persistence was high during the 4-year follow-up period. The proportion of patients who continued (83.1%) was higher than in the 36-month follow-up of the PANGAEA study (70.4%). When annual therapy continuation rates in the first and second year were considered, they were also slightly higher than those in other real-word studies [12,24,25,27,28]. Improvement of clinical outcomes depends highly on prolonged treatment persistence. Patients on oral DMTs have greater treatment persistence [39] and satisfaction [40] than patients on injectable DMTs, and fingolimod has shown the highest treatment persistent rate among other oral DMTs [41,42]. Our study provides evidence that the high continuation rates with fingolimod are sustained up to 4 years, confirming the high treatment persistence with fingolimod previously observed.

### Limitations of the study

We acknowledge that the study has some limitations that should be considered. Firstly, the impact of fingolimod on other relevant outcomes, such as cognition, fatigue, or health-related quality of life was not assessed. Secondly, the number of patients with available MRI data was limited, and therefore, these findings should be interpreted with caution. Lastly, the data reflect the use of fingolimod within Spanish clinical practice and may not be generalizable where patients’ characteristics and the indication for fingolimod use are different from those in the present study.

## Conclusions

Our registry-based data show that the patient profile was similar to other observational studies and that fingolimod was a safe and effective therapeutic option for RRMS patients, regardless of prior treatment. Relapses and radiological activity decreased, and disability stabilized over a 4-year follow-up period. No unexpected safety events were identified, supporting the use of fingolimod in RRMS patients.

## Supporting information

S1 Table Adverse drug reactions for the overall population. (DOCX)

S2 Table Interruption or discontinuation of fingolimod per year. (DOCX)

## Abbreviations:

C: Clínico
H: Hospital
G: General
U: Universitario/Universitari*
