# Supplementary material for: Four-year safety and effectiveness data from patients with multiple sclerosis treated with fingolimod: The Spanish GILENYA registry
Source: PLoS One. 2021 Oct 13;16(10):e0258437. doi: 10.1371/journal.pone.0258437 (PMC8513911; doi:10.1371/journal.pone.0258437)
Supplement: S1 Table — (DOCX) [file pone.0258437.s001.docx]

**S1 Table. Adverse drug reactions for the overall population**

|  | **Overall population (n=697)** | |
| --- | --- | --- |
|  | **Number (%) of patients with adverse reactions** | **Number of adverse reactions** |
| Blood and lymphatic system disorders  Lymphopenia (Grade 4)  Leucopenia | 88 (12.6)  86 (12.3)  2 (0.3) | 97  95  2 |
| Infections and infestations  Urinary tract infection  Zoster herpes  Candida infection  Upper respiratory tract infection  Oral herpes  Pneumonia  Respiratory tract infection  Pyelonephritis  Herpes simple  Cystitis  Cutaneous leishmaniasis  Tinea versicolor  Yeast infection  Acarodermatitis  Pharyngitis  Influenza  Vaginal infection | 39 (5.6)  12 (1.7)  5 (0.7)  5 (0.7)  4 (5.7)  3 (0.4)  2 (0.3)  2 (0.3)  1 (0.1)  1 (0.1)  1 (0.1)  1 (0.1)  1 (0.1)  1 (0.1)  1 (0.1)  1 (0.1)  1 (0.1)  1 (0.1) | 51  16  5  6  4  4  3  2  1  3  2  1  1  1  1  1  1  1 |
| Hepatobiliary disorders  Hypertransaminasemia  Hepatotoxicity  Liver disorder  Toxic hepatitis | 21 (3.0)  15 (2.5)  3 (0.4)  2 (0.3)  1 (0.1) | 22  16  3  2  1 |
| Cardiac disorders  Bradycardia  Atrioventricular block  Palpitations | 12 (1.9)  9 (1.3)  3 (0.4)  1 (0.1) | 13  9  3  1 |
| Investigations  Increased transaminases | 13 (1.9)  12 (1.7) | 14  14 |
| Nervous system disorders  Headache  Dizziness | 9 (1.3)  6 (0.9)  3 (0.4) | 10  7  3 |
| Skin and subcutaneous tissue disorders  Alopecia  Papulopustular rosacea  Psoriasis | 7 (1.0)  5 (0.7)  1 (0.1)  1 (0.1) | 7  5  1  1 |
| General disorders and administration site conditions  Chest pain  Asthenia  Fatigue  Pyrexia | 5 (0.7)  2 (0.3)  1 (0.1)  1 (0.1)  1 (0.1) | 5  2  1  1  1 |
| Gastrointestinal disorders  Sickness  Diarrhea  Dental caries | 4 (0.6)  2 (0.3)  1 (0.1)  1 (0.1) | 4  2  1  1 |
| Neoplasms benign, malignant and unspecified (incl. cysts and polyps)  Basal cell carcinoma  Kidney neoplasia  Lipoma | 3 (0.4)  1 (0.1)  1 (0.1)  1 (0.1) | 3  1  1  1 |
| Eye disorders  Decreased visual acuity  Retinal alteration  Macular edema | 3 (0.4)  1 (0.1)  1 (0.1)  1 (0.1) | 3  1  1  1 |
| Respiratory, thoracic and mediastinal disorders  Dyspnoea | 1 (0.1)  1 (0.1) | 1  1 |
