# Supplementary material for: Four-year safety and effectiveness data from patients with multiple sclerosis treated with fingolimod: The Spanish GILENYA registry
Source: PLoS One. 2021 Oct 13;16(10):e0258437. doi: 10.1371/journal.pone.0258437 (PMC8513911; doi:10.1371/journal.pone.0258437)
Supplement: S2 Table — (DOCX) [file pone.0258437.s002.docx]

**S2 Table. Interruption or discontinuation of fingolimod per year.**

|  | **Year 1** | **Year 2** | **Year 3** | **Year 4** | **Total** |
| --- | --- | --- | --- | --- | --- |
|  | N (%) | | | | |
| Temporary interruption | 22 (3.8) | 19 (4.2) | 11 (3.6) | 3 (1.6) | 43 (6.5) |
| Discontinuation | 35 (6.1) | 42 (9.3) | 43 (14.1) | 24 (12.9) | 106 (15.9) |

*Note:* if a patient had several temporary interruptions during the study follow-up, each temporary interruption was included at each visit. Similarly, discontinuation was registered as ‘permanent discontinuation’ since the first time it was registered onwards. For these reasons, the sum of the number of temporary interruptions and discontinuation at each visit (year 1, year 2, year 3, year 4) does not corresponds to the number of total temporary interruptions and discontinuation reported on the right column (total).
